# Development of Transgenic *Brassica* Crops against Biotic Stresses Caused by Pathogens and Arthropod Pests

**DOI:** 10.3390/plants9121664

**Published:** 2020-11-27

**Authors:** Jorge Poveda, Marta Francisco, M. Elena Cartea, Pablo Velasco

**Affiliations:** Brassica Genetics, Breeding and Biochemistry Group, Biological Mission of Galicia (MBG-CSIC), 36143 Pontevedra, Spain; mfrancisco@mbg.csic.es (M.F.); ecartea@mbg.csic.es (M.E.C.); pvelasco@mbg.csic.es (P.V.)

**Keywords:** *Brassica*, *Brassica napus*, Bt, chitinase, *Sclerotinia sclerotiorum*, *Plutella xylostella*

## Abstract

The *Brassica* genus includes one of the 10 most agronomically and economically important plant groups in the world. Within this group, we can find examples such as broccoli, cabbage, cauliflower, kale, Brussels sprouts, turnip or rapeseed. Their cultivation and postharvest are continually threatened by significant stresses of biotic origin, such as pathogens and pests. In recent years, numerous research groups around the world have developed transgenic lines within the *Brassica* genus that are capable of defending themselves effectively against these enemies. The present work compiles all the existing studies to date on this matter, focusing in a special way on those of greater relevance in recent years, the choice of the gene of interest and the mechanisms involved in improving plant defenses. Some of the main transgenic lines developed include coding genes for chitinases, glucanases or cry proteins, which show effective results against pathogens such as *Alternaria brassicae*, *Leptosphaeria maculans* or *Sclerotinia sclerotiorum*, or pests such as *Lipaphis erysimi* or *Plutella xylostella*.

## 1. Introduction

Taxonomically, the *Brassica* genus belongs to the Brassicaceae family (tribe Brassiceae), which encompasses 338 genera and 3709 species, mostly herbs with annual, biennial or perennial growth habits. They are also known as Cruciferae due to its characteristic flower conformation of four petals arranged in a cross-shape. Brassicaceae species are native to the Irano–Turranian and Mediterranian regions, being distributed in temperate regions. Specifically, *Brassica* is the most prominent genus in the Brassicaceae family and includes 39 species, many of which are cultivated for their edible roots, leaves, stems, buds, flowers, mustard and oilseeds [1].

The principal vegetable species belonging to the *Brassica* genus are *Brassica oleracea* (i.e., broccoli, cabbage, cauliflower, kale, Brussels sprouts, etc.), *Brassica rapa* (i.e., turnip, Chinese cabbage and pak choi), *Brassica napus* (i.e., rapeseed and leaf rape), *Brassica juncea* and *Brassica carinata* (mustards) [2]. During the 1930s, the chromosome number and genetic relationships between the cultivated *Brassica* species was established in a relationship known as “U’s Triangle”. Diploid species *Brassica nigra* (BB, *n* = 8), *B. oleracea* (CC, *n* = 9) and *B. rapa* (AA, *n* = 10) were determined to be the progenitors of allopolyploid species *B. carinata* (BBCC, *n* = 17), *B. juncea* (AABB, *n* = 18) and *B. napus* (AACC, *n* = 19) [1,3].

Crops belonging to the *Brassica* genus are among the 10 most economically important vegetable crops in global agriculture and markets. They are mainly cultivated in temperate regions of the northern hemisphere, such as areas of Europe, the Mediterranean area, Southwestern and Central Asia, China and Japan, and North America. In 2018, the Food and Agriculture Organization of the United Nations (FAO) reported a global production of *Brassica* crops close to 135 million tons, being 26.5 million tons of ‘cauliflowers and broccoli’ in almost 1.5 million ha, 75 million tons of ‘rapeseed’ in more than 37.5 ha, and about 70 million tons of ‘cabbages and other *Brassica* crops’ from almost 2.5 million ha in more than 150 countries [4].

Due to their wide adaptation and ability to thrive under varying agroclimatic conditions, *Brassica* crops are grown throughout the world for food, animal forage and fodder and also for industrial applications [5]; they also have an allelopathic use for sustainable agriculture [6] and are grown as phytoremediators against heavy metals such as cadmium [7]. As far as food is concerned, nowadays, consumers are demanding products that are rich in nutrients for optimal health benefits. In this respect, the popularity of *Brassica* products is increasing because of their nutritional value, and anticancer, antioxidant and anti-inflammatory properties. Nutritionally, these vegetables are low-fat, have a high vitamin (C and E) content and contain minerals (P, S, Cl, Ca, Fe, Sr, K, Cr, Mn, Se and Zn) and fiber. In addition, they contain important phytochemicals that are beneficial for human health, such as anthocyanins, flavonoids, terpenes, S-methyl cysteine sulfoxide, coumarins and other small compounds [8]. However, the most characteristic compounds are glucosinolates, which are a group of secondary metabolites that are only present in Brassicaceae and immediate families. They have various functions within the plant, being especially important in the defense against pathogens and herbivores [9].

## 2. *Brassica* Transgenesis

Currently, the main strategies for the improvement of *Brassica* species are molecular breeding and genetic transformation technology. The development of different genetic engineering tools has opened up vast opportunities for the introduction of novel and useful genes of agronomical importance. In this sense, many agronomical important genes have been identified and transferred into *Brassica* species [10]. Transgenic lines developed so far in the *Brassica* genus are focused on tolerance to salt stress [11], phytoremediation of heavy metals [12], oil quality improvement, insect resistance, herbicide resistance, development of male-sterile lines and the production of pharmacological and industrial products [10].

The genetic transformation of *Brassica* has been carried out in almost all the economically important species, but far more work has been conducted with *B. napus* (rapeseed or canola) than with any of the others. In *B. rapa*, both oilseed and Chinese cabbage forms have been transformed. *B. oleracea* transgenic plants include all the major vegetables like cabbage [13], broccoli [14], Brussels sprouts and cauliflower. A few reports dealing with *B. nigra*, *B. juncea* and *B. carinata* have also been conducted.

A fundamental step in the development of transgenic lines is regeneration, which has been achieved in *Brassica* lines by somatic embryogenesis and organogenesis using different explants. As far as transformation methods are concerned, the *Agrobacterium*-mediated gene uptake has been most commonly used for *Brassica* [10]. With regard to *Agrobacterium tumefaciens*, effective transformations in cotyledonary or hypocotyl explants of Chinese cabbage [15,16], or by floral dip in rapeseed [17] have been reported. In addition, there are perfectly developed protocols for this from seedling explants [18], even having developed an effective transient expression system [19], and also with *Agrobacterium rhizogenes* [20]. However, nowadays, methods are beginning to be developed with other vector bacteria, such as the rhizospheric bacterium of rapeseed *Ensifer adhaerens*, which is capable of genetically transforming its original host [21].

An aspect that is always linked to the development of transgenic crops is their safety for the environment, which is based on the capacity of new crops to survive outside the agro-system and the potential exchange of genetic information from these crops to related plants. This is particularly relevant in the case of *B. napus* because of its known potential for genetic exchange with wild or weedy relatives. In this sense, it has been reported how a glyphosate-resistant rapeseed line, which has been cultivated in Australian fields since 2009, is able to grow naturally outside the agro-system. However, it was also shown how it ends up becoming extinct from the area within 3 years, without being able to become an invasive plant [22]. On the other hand, there are numerous examples of hybridizations between various transgenic crops of the genus *Brassica* and nearby wild species, such as *B. rapa*, *B. juncea*, *B. oleracea*, *B. nigra*, *Hirschfeldia incana* and *Raphanus raphanistrum*. The recombination of sets of different chromosomes may make not all hybrids viable, but there are reports of hybrids showing good field performance, although they produce fewer seeds than their wild parent [23].

## 3. List of Common Pests and Pathogens of *Brassica* Species

It is estimated that pathogens and pests produced by insects and mites are capable of causing losses in the *Brassica* crop production of up to 50–60% and in quality, which result in substantial economic losses [24,25,26]. Although biotic stresses may vary depending on the geographical area and the particular crop, there are several of them that are common to many of the different species and varieties grown. Below, we will delve into those *Brassica* crop pathogens and pests against which effective transgenic lines have been developed.

Regarding bacterial diseases, *Xanthomonas campestris* pv. *campestris* (Xcc), the causal agent of black rot, is considered to be one of the most important pathogens affecting *Brassica* vegetables worldwide. There are nine races of Xcc, but races 1 and 4 are considered the most virulent and widespread [27]. Xcc penetrates leaves through hydathodes or wounds. Subsequently, the pathogen travels through the vascular system, thus invading the xylem and colonizing the mesophyll. This causes the appearance of typical symptoms, which include V-shaped chlorosis from the edges of the leaves, necrosis and darkening of leaf veins and the stem vascular tissue [28]. As the disease progresses, wilting and necrosis throughout the plant appear. When the pathogen spreads along the veins and petioles to the plant stems and roots, the vascular tissue within the roots and stems turns black, and the whole plant dies, sometimes accompanied by a secondary soft rot [29]. On the other hand, the most common disease of Brassicaceae is soft rot, mainly caused by highly pectinolytic bacteria *Pectobacterium carotovorum* (Pc) (formerly *Erwinia carotovora*). Pc may infect the plant through natural openings or wounds caused by insects, other diseases, or by abiotic factors, such as low temperatures or mechanical damages. For example, symptoms of bacterial soft rot on cabbage include water-soaked lesions, which later become a rotted mass of macerated tissue [30].

As far as fungal diseases are concerned, the most widely distributed pathogen in all *Brassica* crops are the four species described of genus *Alternaria*: *Alternaria alternata*, *Alternaria brassicae*, *Alternaria brassicicola* and *Alternaria raphani*. *A. brassicae* is a major pathogen of oil-yielding *Brassica*, while the other three are more common on vegetable crops. All the four *Alternaria* species cause symptoms on cotyledons at the seedling stage and on leaves, leaf petiole, stem, inflorescence, siliquae and seeds at the adult stage. Quantitative and qualitative losses in the yield of oil seeds and vegetables range from 11 to 100%, depending on the time of infection, prevailing environmental conditions after infection and the strategies used for its control [31].

Sclerotinia stem rot, caused by *Sclerotinia sclerotiorum* (Ss) fungi, is one of the most devastating diseases for *B. napus* and *B. juncea* worldwide, including Australia, China, Europe and North America. In Australia, this disease causes up to 24% yield loss. The pathogen attacks cotyledons and leaves in seedlings and stems and leaves in adult plants, causing some key symptoms like water-soaked lesions, necrotic tissues with fluffy white mycelium and sclerotia inside of stems [32]. In rapeseed, a major constraint in production is blackleg disease, caused by ascomycete fungus *Leptosphaeria maculans* (Lm). Blackleg has been reported in all canola-growing regions except for China, and causes annual yield losses of 10–20%. In seedlings, the fungus colonizes intercellular spaces, thus causing necrotic cotyledon and leaf lesions before entering a biotrophic phase whilst growing down the petiole and into the stem. At plant maturity, the fungus causes blackening of the stem and cankering, thus restricting the nutrient flow up the plant, and in severe situations, completely killing the plant [33].

Within the group of oomycete pathogens, *Albugo candida* (Ac) is a biotrophic plant pathogen that causes white blister rust disease in Brassicaceae. Zoospores enter their host through stomata, where they germinate and begin colonization of mesophyll cells. Finally, the oomycete forms zoosporangia that appear as white pustules rupturing the epidermis, hence constituting the visible symptoms of the disease [34]. Additionally, in regard to affecting the aerial part of the plant, we can find the disease known as downy mildew, which is caused by oomycete *Hyaloperonospora brassicae* (Hb) (formerly *Peronospora parasitica*). This pathogen causes destructive damages on *Brassica*, *Raphanus* and *Sinapis* species, including on many economically relevant crops, such as broccoli, cabbage, radish, rapeseed, tatsoi and wasabi. In *Brassica* seedlings, it appears on cotyledons and leaves as a pale green, yellowish growth on leaf undersides, while it causes irregular angular yellow blotches in older plants, which may have dark speckling [35,36].

Turnip mosaic virus (TuMV), family Potyviridae, causes a damaging disease in many kinds of *Brassica* and other crops worldwide, which results in losses in yield and quality of produce. TuMV is transmitted nonpersistently by more than 40 different aphid species, having a wide natural host range. The disease symptoms often include vein clearing, mosaic, necrosis, plant stunting and plant death, although they depend on the TuMV strain, host plant and environmental conditions [37].

Regarding insect-pests, aphids represent a major constraint to the production of many crops worldwide, especially *Brassica* crops. Mustard or turnip aphid *Lipaphis erysimi pseudobrassicae* (Lep) is one of the most destructive pests for *Brassica*, causing over 50% yield loss. These aphids feed by sucking sap from their host plants, which leads to stunted growth, and they excrete honeydew, which leads to fungal growth (sooty mold). Furthermore, aphids of the *L. erysimi* group transmit over 13 different viruses, including important viruses of the Brassicaceae, such as TuMV [38].

The Lepidopteran pest, diamondback moth, *Plutella xylostella* (Px), is the most destructive insect pest for *Brassica* crops. Larvae feed on host plants’ leaves, causing substantial crop losses [39]. Similarly, the large white butterfly, *Pieris brassicae* (Pb), also known as cabbage white butterfly, can be very destructive, and up to 90% loss in yield of certain *Brassica* spp. has been reported, especially in developing countries where chemical insecticides are too expensive to be used regularly against this pest [40].

## 4. Recent Progress of Transgenic Research in *Brassica* Species against Pathogens

There has been a large number of transgenic *Brassica* crops developed against pathogen diseases so far, thanks to a wide variety of different genes from very diverse organisms. Table 1 contains the information on the transgenic lines developed, and indicates information such as the target pathogen or the mechanism involved in resistance.

The greatest number of transgenic lines of *Brassica* crops against fungal pathogens was made by transformation with genes that code for chitinases. Chitinase is an enzyme (a pathogenesis-related protein) that catalyzes the degradation of chitin, which is the main component of the fungal cell wall. In this sense, the most used transformation methodology is from hypocotyls using *A. tumefaciens*, as it was done in *B. juncea*, which was transformed with the gene that encodes chitinase C (*ChiC*) from *Streptomyces griseus* [88].

Different species belonging to the *Trichoderma* genus are widely studied and used as biocontrol agents in agriculture due to their ability to parasitize different fungal pathogens, by degrading their cell wall through the release of powerful chitinases [93]. This machinery has been used as a resource in the transformation of *Brassica* crops, thus obtaining plants that are resistant to *Alternaria brassicicola* [58,59] and *A. brassicae* [55] thanks to the endochitinase 42 from *Trichoderma harzianum* and *T. virens*. Transgenic lines showed a delayed onset of lesions, as well as a 30–73% reduction in the infected leaf area compared to non-transformed plants. In addition, this chitinolytic effect is increased when a chimeric chitinase produced by the fusion of the *Chit42* gene from *Trichoderma atroviride* and a chitin-binding domain from *Serratia marcescens* is used, hence causing a great inhibition of fungal growth [61,82].

Other organisms have been used to obtain genes that encode chitinases, such as class I chitinase from tobacco in *B. juncea* against *A. brassicae*, which inhibited the fungal colony size by 12–56% over the nontransgenic control [49]. The *NIC* gene has even been used, coding for a synthetic chitinase, in *B. juncea* transformation, for which leaf tissue extracts showed considerable resistance and antifungal activity against *A. brassicae* [60]. We can also mention a double transformation of *B. juncea* with two genes from barley, class II chitinase and the gene coding for a ribosome inactivating protein (*RIP*), which inactivates foreign ribosomes in distantly related species and in other eukaryotes, including fungi [51].

Other double transformations with chitinases and other genes have been reported to perform important antifungal activities. Against *S. sclerotiorum*, transgenic lines of *B. napus* have been developed that coexpress the *Chit42* gene from *T. atroviride* and a defensin from *Raphanus sativus*, involved in increasing the fungal membranes permeability [83], or a polygalacturonase inhibiting protein 2 from *Phaseolus vulgaris* (a protein inhibiting fungal endo-polygalacturonases) [84], showing greater resistance to the disease thanks to a delayed onset and to the restricted size and expansion of lesions when compared to wild type plants. We can also see the chitinase I gene from *Paecilomyces javanicus* together with a sporamin gene from sweet potato, which is a protein involved in trypsin inhibition [76]. However, in isolation, these genes are also capable of improving the defensive capacity of *Brassica* crops against different pathogens. For example, the defensive capacity of *B. napus* against *S. sclerotiorum* is improved by the transformation with a defensin gene from *Orychophragmus violaceus* [74] or with a polygalacturonase inhibiting protein 2 gene (*PGIP2*) from *Oryza sativa* [85]. *PGIP2* genes are not only effective against fungal polygalacturonases, as it has also been observed in the transformation of *B. napus* with a *PGIP2* gene from *Proteus vulgaris* against *Rhizoctonia solani*, but they inhibit the disease up to 37% [70]. They are also effective against bacterial polygalacturonases, as in the over-expression of the *PGIP2* gene from Chinese cabbage in the same plant, hence exhibiting improved resistance to bacterial soft rot caused by Pc (up to 54%) [45].

Other examples of transformations of *Brassica* crops with genes that code for defense-related enzymes are found in glucanases, lipases or oxalate oxidases. Since β-1, 3-glucan is a structural polymer present in the fungal cell wall, among fungal resistant genes, glucanases that are potential antifungal agents thanks to their glucan degradation activity are excellent candidates for controlling fungal pathogens development. In this sense, transformation of *B. napus* with a β-1,3-glucanase from *Trichoderma virens* showed a stronger inhibition against *S. sclerotiorum* hyphal growth [80], and the transformation of *B. juncea* with the class I basic glucanase from tomato showed a restricted number, size and spread of lesions caused by *A. brassicae* [50]. Similarly, lipases act against the cellular membrane of pathogenic fungi. In *B. napus*, lipase GDSL1 from *Arabidopsis thaliana* was characterized as an extracellular GDSL lipase functioning in *S. sclerotiorum* resistance. By releasing fragments of the fungal membrane, such as phosphatidic acid, GDSL1 activates the defensive responses of the plant through an increase in reactive oxygen species (ROS) and salicylic acid (SA) levels. However, this plant defense response could also be a consequence of the release of phosphatidic acid from the plant plasma membrane, instead of the fungal plasma membrane [86]. During plant infection, *S. sclerotiorum* secretes oxalic acid, thus acidifying the plant tissue surrounding the site of infection and causing tissue damage, chelating divalent cations and sequestration of calcium that may weaken cell walls, and inhibiting the plant defense route by phenolic compounds. By transforming *B. napus* plants with a coding gene for enzyme oxalate oxidase from wheat, the breakdown of oxalic acid to CO_2_ and H_2_O_2_ was achieved, and also a significant reduction in infection caused by the pathogenic fungus [72].

In recent decades, there has been an increased interest in producing transgenic plants that are strongly resistant to a broader spectrum of microbial phytopathogens through the expression of cationic antimicrobial peptides (CAPs). Although CAPs are structurally diverse, most of them fall into two general structure types: α-helical peptides, such as cecropins and magainins, and β-sheet peptides, such as defensins, protegrins, and tachyplesins [94]. In *Brassica* crops, there are several transformations with genes coding for this type of protein, which have resulted in important inhibitions of pathogen growth and the development of the disease, thanks to a powerful membrane antagonism. Regarding α-helical peptides, we found effective rapeseed transgenic lines against *L. maculans* [66], the transformation of *B. juncea* with the gene coding for a cecropin-melittin cationic peptide has been effective against *A. brassicae* and *S. sclerotiorum* [53], and also *B. napus* with a magainin II analogue against *S. sclerotiorum* [79]. The simultaneous transformation of cauliflower with a cecropin from *Antheraea polyphemus* and a magainin from *Xenopus laevis* against Xcc [46] is effective as well. Another group of antimicrobial proteins is found in those known as lipid transfer proteins, which also cause a powerful membrane antagonism against different pathogens. Transgenic lines of *B. napus* that overexpress these type of genes from *Leonurus japonicus* and *Oryza sativa* show greater resistance against *S. sclerotiorum*, as shown in in vitro experiments with crude leaf extracts from transgenic lines, which significantly inhibit mycelial growth [77,78].

Although *Brassica* crops have been transformed with genes directly involved in defensive responses against pathogens, they have also been transformed with intermediaries in the signaling of plant responses. Once the plant recognizes the pathogen attack, it activates the plant defense responses through a signaling cascade, where mitogen-activated protein kinases (MAPKs) play a key intermediate role [95]. In *Brassica* crops, overexpression of MAPKs genes, such as MPK3 and MPK4, increases the resistance of *B. juncea* and *B. napus* against *A. brassicae* and *S. sclerotiorum* through the activation of the SA- and JA-mediated plant-defenses, respectively [57,73]. WRKY transcription factors are a type of DNA-binding proteins, whose domain is defined by the conserved amino acid sequence WRKYGQK. They are activated by MAPKs and are responsible for activating the gene expression related to the response to SA and JA pathogens [95,96]. Overexpression of *WRKY33* in *B. napus* has shown significant increases in plant resistance against *S. sclerotiorum* [81]. On the other hand, the non-expressor of pathogenesis-related genes 1 (*NPR1*) is a master regulator of SA-mediated systemic acquired resistance (SAR), which is a broad-spectrum disease resistance mechanism in plants [95,97]. *NPR1* overexpression in *B. juncea* and *B. napus* led to an increase in the SA-mediated defensive response and a greater resistance against *A. brassicae*, *E. polygoni* and *S. sclerotiorum* [56,87].

Against viruses, one of the most widely used mechanisms in plant transgenesis is the use of RNA interference that blocks the pathogen molecular machinery. By analyzing a fragment of a 130 bp coat protein, it was possible to develop an efficient hairpin construct against TuMV. The transformation of *B. napus* with this construction assumes the total absence of viral infection [41].

On the other hand, *Brassica* crops are not only a sink for genes from other organisms, but they are also used as a source of genes for improving the resistance of other crops against pathogens and pests. In tobacco, the ectopic expression of an annexin from *B. juncea* confers enhanced resistance against pathogenic oomycete *Phytophthora parasitica* var. *nicotianae*, thanks to an increase in message levels for several pathogenesis-related proteins [98]. Additionally, in rice, the expression of *B. juncea nonexpressor of pathogenesis-related 1* (*BjNPR1*) gene, exhibits enhanced resistance against fungi *Magnaporthe grisea* and *Rhizoctonia solani*, and bacteria *Xanthomonas oryzae* pv. *oryzae* [99]; resistant plants have also been obtained against this bacteria pathogen thanks to transformation with the *Brassica rapa cysteine protease 3* (*BrCP3*) gene [100]. Additionally, in peanut, the co-overexpression of *B. juncea NPR1* (*BjNPR1*) and *Trigonella foenum-graecum defensin* (*Tfgd*) provides protection against *Aspergillus flavus* and *Cercospora arachidicola* [101].

## 5. Recent Progress of Transgenic Research in *Brassica* Species against Arthropod Pests

Genes used in the transformation of *Brassica* crops for resistance against pests are less diverse than in the case of defense against pathogens. Although almost all of the transgenic lines developed to date against insects are *Bacillus thuringiensis* (Bt) crops, there are other examples that share the strategies used against pathogens, which are represented in Table 2. Information such as the target arthropod pest or the mechanism involved in resistance is reflected. As with viruses, the use of interfering RNA has shown good results in controlling herbivorous arthropods. The transformation of Chinese cabbage with complementary fragments of the gene encoding coatomer protein complex subunit 2 (*COPB2*) derived from mite *Tetranichus urticae* was achieved. A high mite resistance with nearly a 100% mortality rate was reported. This study demonstrated the effectiveness of the plant-mediated RNAi technique in developing mite-resistant plants [102].

One of the most important direct defense responses in plants against the attack of phytophagous insects is the production of insecticidal peptides or proteins. Among the entomotoxic proteins, we can find the group of carbohydrate-binding proteins or lectins. These proteins, after being ingested by phytophagous insects, are released from disrupted cellular structures and come into contact with carbohydrate structures present in the midgut of insects [146]. In the development of transgenic lines with lectins, there are several examples of the control of aphid *Lipaphis erysimi* by means of the blockage of the insect gut epithelial membrane and the inhibition of nutrient uptake. In this sense, genes coding for agglutinins (from wheat, garlic or onion) have been the most used in the transformation of *B. juncea*, reporting a high mortality and significantly reduced fecundity of aphids [103,104,105]. In conjunction with a lentil lectin, the transformation of *B. juncea* with a chickpea protease inhibitor was achieved, thus reducing aphid survival by 40%. This is because the protease inhibitors of plant origin are basically competitive inhibitors, acting as pseudo-substrates that bind to the active site of respective proteases, thus causing a disruption in assimilation of dietary protein in herbivorous insect pests, which primarily delays their significant growth and development [107].

Sporamins is a widely used group in inhibitor protease transgenesis, and in the transformation of Chinese cabbage [136] and rapeseed against the diamondback moth (*Plutella xylostella*), in conjunction with chitinase 1 from *Paecilomyces javanicus* [76]. The cuticle of insect species consists largely of chitin. Therefore, chitinase production has been used as a criterion for selecting potential biocontrol agents against insects. Therefore, because of their ability to interfere with chitin deposition, microbial chitinolytic enzymes have been considered to be important in the biological control of many insects [147]. However, in the transformation of *B. napus* against *P. xylostella*, chitinases from Lepidoptera have also been used, such as *Manduca sexta*, together with the gene encoding for a neurotoxin of contractive paralysis type insect-specific from scorpion *Buthus martensii* [127].

*Bacillus thuringiensis* (Bt) is the main microorganism used in the biological control of insect pests. Bt produces a variety of entomotoxic crystalline proteins (Cry) known as δ-endotoxins. When ingested by susceptible insects, crystals dissolve in the insect gut and protoxins are liberated and activated proteolytically to a toxic fragment. This fragment binds to a specific cadherin on the brush border membrane of gut epithelial cells, and inserts into the membrane, thus generating pores. The change in membrane permeability leads to a colloid osmotic lysis of the gut epithelial cells and, ultimately, to insect death. In the last 30 years, numerous transgenic crops expressing Bt insecticidal crystals have been developed. The potential benefits of Bt crops include increased crop yields, a reduction in the use of broad-spectrum insecticide and the associated application costs and energy input, a reduced need for scouting, an improvement of the health conditions of farm workers, and savings in time [148]. In *Brassica* crops, transgenic-Bt lines of the most cultivated species have been developed against Lepidoptera-pests, such as *P. xylostella*, *Trichoplusia ni*, *Pieris rapae* and *Helicoverpa armigera* (Table 2), which showed significant resistance. Moreover, it has been proven that the presence of cry genes in the genome of *Brassica* crops does not modify the synthesis or mode of action of constitutive and herbivore-inducible glucosinolates, hence maintaining an important strategy for these crops against herbivores [130].

## 6. Progress on Transgenic Research in Beneficial Biotic Interactions of *Brassica* Species

Since the first transgenic field, about 25 years ago, different studies have attempted to understand the potential negative effects of this new technology on the environment. A fundamental part is the effect that they can have on different beneficial organisms (soil microorganisms, pollinating insects, etc.) [149].

As far as rhizospheric microorganisms are concerned, studies have only been carried out with the most widely cultivated transgenic *Brassica* in the world, which is *B. napus*. Transgenic *B. napus* harboring the synthetic chitinase (*NiC*) gene exhibits broad spectrum antifungal resistance, for example against *Alternaria brassicicola*. Since rhizosphere microorganisms play an important role in element cycling and nutrient transformation, biosafety assessment of *NiC* containing transgenic plants on the soil ecosystem is a regulatory requirement. By analyzing the rhizosphere enzyme activities and the microbial community structure, it was demonstrated how *NiC B. napus* lines may not affect rhizospheric microorganisms [150]. The effects produced by herbicide-tolerant transgenic lines must be addressed in a different way, since the application of an herbicide must be analyzed as an indirect effect of the use of a transgenic line. In this sense, by Illumina MiSeq sequencing method, the effect of transgenic glufosinate-tolerant rapeseed and the associated herbicide application on rhizospheric bacterial communities were studied. The results showed that growing glufosinate-resistant transgenic rapeseed line Z7B10 and the application of glufosinate herbicide had no adverse effects on the rhizospheric bacterial community composition [151].

Moreover, several studies have been carried out in soils contaminated with heavy metals in order to determine potential modifications in the beneficial interaction between *B. napus* and plant growth promoting bacteria (PGPRs). A transgenic rapeseed line that overexpresses the gene for enzyme 1-aminocyclopropane-1-carboxylate (ACC) deaminase from *Pseudomonas putida* inoculated with the *P. putida* bacterium, developed better than its nontransgenic control in nickel-contaminated soils. This shows that the interaction between *P. putida* and transgenic *B. napus* leads to greater plant growth and higher heavy metal tissue accumulation [152,153].

In *B. napus*, the ability of different species of the fungal genus *Trichoderma* to increase their productivity [154] and tolerance against abiotic stresses such as drought or salinity has been described [155]. In rapeseed plants transformed with the *Thkel1* gene from *T. harzianum*, which codes for a Kelch domain protein, it is pointed out how this protein can modify glucosinolate hydrolysis by binding myrosinase enzymes, hence preventing the formation of toxic compounds such as isothiocyanates. As a consequence, the transgenic rapeseed plants in interaction with *T. harzianum* significantly increased the levels of fungal root colonization and their productivity. In addition, the presence of this Kelch domain protein reported increases in the β-glucosidase activity, which implies a greater resistance against foliar infection by *L. maculans* in rapeseed plants [69].

On the other hand, the potential detrimental effects of Bt transgenic lines against non-target arthropods has been widely covered in current reviews [156,157]. The potential detrimental effects of the interaction of transgenic *Brassica* lines with insects and other beneficial arthropods have been studied in rapeseed and Chinese cabbage. In rapeseed Bt transgenic lines, it has been reported that there could be an exposure to the insecticidal protein of transgenic lines by diurnal pollinators Lepidoptera, as larvae (mainly of Pieridae) through the host and through pollen, and as adults via pollen and nectar. This could lead to a localized loss of biodiversity that requires exhaustive environmental risk assessments [158]. Similarly, the effects of silkworm (*Bombyx mori*) larvae with pollen from Bt Chinese cabbage have been studied. A decrease in the survival rate and body weight of *B. mori* larvae fed only with Bt pollen was observed, which was an indication of the negative effect that Bt Chinese cabbage in the field can have on pollinators. However, the authors point out how the reduced interaction of the bumblebee with Chinese cabbage pollen in the field reduces the actual risk to a minimum [159]. Nevertheless, other transgenic lines against pest insects such as rapeseed, which overexpresses pea lectin, have been used to feed bee larvae (*Apis mellifera*) with their pollen, and it did not show any negative effect on larval mortality, weight or development time [160]. Regarding the effect on herbivore predators that feed on Bt *Brassica* crops, a study has been carried out with the wolf spider (*Pardosa astrigera*). After feeding fruit flies (*Drosophila melanogaster*) with Bt cabbage, it was found that the insecticidal protein had no effect on them, but it did accumulate inside their bodies. The wolf spider was subsequently fed with these fruit flies, not quantifying any negative effect on the predator [161].

## 7. Conclusions

The present work compiled the existing studies to date on the interaction between transgenic plants and biotic factors, focusing on those of greater relevance in recent years, the choice of the gene of interest and the mechanisms involved in increasing plant defenses. In the improvement of these crops, numerous transgenic lines have been developed, showing characteristics such as greater tolerance to abiotic stresses, greater phytoremediation capacity or capability of synthesizing and accumulating compounds of industrial interest. In this sense, as a consequence of the importance of pathogens and pests in *Brassica* crops cultivation (losses of up to 60%), numerous resistant transgenic lines have been developed in recent years.

The transformation of *Brassica* crops with chitinases has reported numerous lines that are resistant to fungal pathogens in an effective way. Against bacteria and fungi, the transformation with antimicrobial peptides, such as defensins, represents a very effective strategy in the control of these pathogens. On the other hand, modification of plant defense responses through transformation with genes involved in intermediate steps between the recognition of the pathogen and the defensive response has reported significant reductions in the development of diseases in *Brassica* transgenic crops.

In the development of resistant lines against insect pests, cry genes from Bt are the most widely used, although there are other genes used in *Brassica* crops. Based on their ability to block intestinal activity, carbohydrate-binding proteins or lectins have been used with significant reductions in the attack of Hemipteran and Lepidopteran pests.

Regarding the interactions of transgenic lines of *Brassica* crops with beneficial microorganisms from the agro-system, effects of an increasing mutualistic interaction have been reported, without any negative effects being described for the moment. In this sense, it is noteworthy that only *B. napus* has been used in this type of study, and there is still much to know. On the other hand, the interaction with insects does not fully confirm the absence of negative effects on the natural enemies of pest insects or on pollinators. The development of many other studies is needed, since, until now, only investigations with cabbage and Chinese cabbage exist.

## Figures and Tables

**Table 1:** Transgenic *Brassica* crops against pathogens.

Pathogen		*Brassica* Species	Gene			Mechanism	Reference
Group	Species		Name	Protein	Origin		
Viruses	Turnip Mosaic Virus	*B. napus*	*CP*	Coat protein	Turnip Mosaic Virus	RNA silencing mechanism	[41]
Bacteria	*Pectobacterium carotovorum* ssp. *carotovorum*	*B. rapa* ssp. *pekinensis*	-	Anti-bacterial peptide	Not indicated	Bacteriostasis action	[42]
		*B. rapa* ssp. *pekinensis*	*BAA1*	Bromelain 1	Pineapple	Plant programmed cell death	[43]
		*B. rapa* ssp. *pekinensis*	*PinII*	Proteinase inhibitor II	Potato	Inhibition of bacterial cell communication.	[44]
		*B. rapa* ssp. *pekinensis*	*BrPGIP2*	Polygalacturonase-inhibiting protein 2	*B. rapa ssp. pekinensis*	Inhibition of bacterial polygalacturones	[45]
	*Xanthomonas campestris* pv. *campestris*	*B. oleracea* var. *botrytis*	-	Cecropin B	*Antheraea polyphemus*	Lyse bacterial cell membranes	[46]
			-	Magainin II	*Xenopus laevis*	Disruption of microbe membranes	
	*Ralstonia solanacearum*	*B. rapa*	*RRS1*	*R. solanacearum* resistance protein 1	*Arabidopsis thaliana*	Activation of defensive response by JA/ET route	[47]
			*RPS4*	*Pseudomonas syringae* resistance protein 4			
Fungi	*Alternaria brassicae*	*B. juncea*	-	Lectin	*Hevea brasiliensis*	Fungal cell wall carbohydrate binding (immobilization)	[48]
		*B. juncea*	-	Class I chitinase	Tobacco	Fungal cell wall degradation	[49]
		*B. juncea*	-	Class I basic glucanase	Tomato	Fungal cell wall degradation	[50]
		*B. juncea*	-	Class II chitinase	Barley	Fungal cell wall degradation	[51]
			*RIP*	Ribosome inactivating protein		Inactivation of foreign ribosomes in distantly related species and in other eukaryotes, including fungi	
		*B. napus*	*PmAMP1*	Cysteine rich antimicrobial peptide 1	*Pinus monticola*	Unidentified	[52]
		*B. juncea*	*MsrA1*	Cecropin–melittin cationic peptide	-	Powerful membrane antagonism	[53]
		*B. juncea*	-	Lectin	Chickpea	Fungal cell wall carbohydrate binding (immobilization)	[54]
		*B. juncea*	*ech42*	Endochitinase 42	*Trichoderma virens*	Fungal cell wall degradation	[55]
		*B. juncea*	*NPR1*	Non-expressor of pathogenesis-related	*B. juncea*	Activation of the SA-mediated plant-defense	[56]
		*B. juncea*	*MPK3*	Mitogen-activated protein kinase 3	*B. juncea*	Activation of the SA-mediated plant-defense	[57]
	*A. brassicicola*	*B. oleracea* var. *italica*	-	Endochitinase	*Trichoderma harzianum*	Fungal cell wall degradation	[58,59]
		*B. juncea*	*ech42*	Endochitinase 42	*Trichoderma virens*	Fungal cell wall degradation	[55]
		*B. juncea*	*NIC*	Synthetic chitinase	-	Fungal cell wall degradation	[60]
	*A. solani*	*B. napus*	*Chit42*	Endochitinase 42	*Trichoderma atroviride*	Fungal cell wall degradation	[61]
	*Botrytis cinerea*	*B. napus*	*MAM1*	Methylthioalkylmalate synthase 1	*B. napus*	Glucosinolate biosynthesis	[62]
			*CYP83A1*	Cytochrome P450 83A1			
			*UGT74B1*	Glucosyltransferase 74B1			
	*Erysiphe polygoni*	*B. napus*	*katE*	Catalase E	*Escherichia coli*	Control of the activation of plant defense responses through the H_2_O_2_ dismutation	[63]
		*B. juncea*	*NPR1*	Non-expressor of pathogenesis-related	*B. juncea*	Activation of the SA-mediated plant-defense	[56]
	*Fusarium oxysporum*	*B. napus*	*Chit42*	Endochitinase 42	*Trichoderma atroviride*	Fungal cell wall degradation	[61]
	*Leptosphaeria maculans*	*B. napus*	*Cf9*	*Cladosporium fulvum* resistance protein 9	Tomato	Hypersensitive response activation	[64]
			*Avr9*	Dominant pathogen avirulence protein 9	Tomato	Hypersensitive response activation	
		*B. napus*	*DRR206*	Dirigent protein	*Pisum sativum*	Unidentified	[65]
		*B. napus*	*MiAMP1*	Antimicrobial peptide 1	*Macadamia integrifolia*	Powerful membrane antagonism	[66]
		*B. napus*	*Lm1*	L. maculans 1	*B. nigra*	Unidentified	[67]
		*B. napus*	*PmAMP1*	Cysteine rich antimicrobial peptide 1	*Pinus monticola*	Unidentified	[52]
		*B. napus*	*DWF4*	C-22 hydroxylase	*A. thaliana*	Brassinosteroids biosynthesis	[68]
		*B. napus*	*Thkel1*	Kelch domain protein	*Trichoderma harzianum*	β-glucosidase activity	[69]
	*Peronospora p* *arasitica*	*B. napus*	*katE*	Catalase E	*E. coli*	Control of the activation of plant defense responses through theH_2_O_2_ dismutation	[63]
	*Rhizoctonia solani*	*B. napus*	*DRR206*	Dirigent protein	*P. sativum*	Unidentified	[65]
		*B. napus*	*pgip* *2*	Polygalacturonase-inhibiting protein 2	*Proteus vulgaris*	Inhibition of fungal endo-polygalacturones	[70]
		*B. napus*	*Chit42*	Endochitinase 42	*Trichoderma atroviride*	Fungal cell wall degradation	[61]
	*Sclerotinia sclerotiorum*	*B. napus*	-	Chitinase	*B. napus*	Fungal cell wall degradation	[71]
			-	Beta-1,3-glucanase	*B. napus*	Fungal cell wall degradation	
		*B. napus*	*DRR206*	Dirigent protein	*P. sativum*	Unidentified	[65]
		*B. napus*	*OXO*	Oxalate oxidase	Wheat	Breakdown of the oxalic acid produced by the fungus.	[72]
		*B. napus*	*MPK4*	Mitogen-activated protein kinase 4	*B. napus*	Activation of the JA-mediated plant-defense	[73]
		*B. napus*	*Ovd*	Defensin	*Orychophragmus violaceus*	Permeabilization of fungal membranes	[74]
		*B. napus*	*scFv*	*S. sclerotiorum* antibody	*S. sclerotiorum*	Binding to the cell wall (immobilization, activation of plant defenses, etc.)	[75]
		*B. napus*	*PjChi-1*	Chitinase 1	*Paecilomyces javanicus*	Fungal cell wall degradation	[76]
			-	Sporamin	Sweet potato	Proteases inhibition	
		*B. napus*	*PmAMP1*	Cysteine rich antimicrobial peptide 1	*Pinus monticola*	Unidentified	[52]
		*B. napus*	*LTP*	Lipid transfer protein	*Oryza sativa*	Powerful membrane antagonism	[77]
		*B. napus*	*LJAMP2*	nsLTPs-like antimicrobial protein	*Leonurus japonicus*	Powerful membrane antagonism	[78]
		*B. napus*	*MSI-99m*	Magainin II analogue	*Xenopus laevis*	Powerful membrane antagonism	[79]
		*B. napus*	*bgn* *13.1*	β-1,3-glucanase	*Trichoderma virens*	Fungal cell wall degradation	[80]
		*B. juncea*	*MsrA1*	Cecropin–melittin cationic peptide	-	Powerful membrane antagonism	[53]
		*B. napus*	*WRKY33*	Protein containing WRKY zinc-finger motifs 33	*B. napus*	Activation of the SA- and JA-mediated plant-defense	[81]
		*B. napus*	*Chit42*	Endochitinase 42	*Trichoderma atroviride*	Fungal cell wall degradation	[82]
		*B. napus*	*Chit42*	Endochitinase 42	*Trichoderma atroviride*	Fungal cell wall degradation	[83]
			-	Defensin	*Raphanus sativus*	Permeabilization of fungal membranes	
		*B. napus*	*Chit42*	Endochitinase 42	*Trichoderma atroviride*	Fungal cell wall degradation	[61]
		*B. napus*	*Chit42*	Endochitinase 42	*Trichoderma atroviride*	Fungal cell wall degradation	[84]
			*PG1P2*	Polygalacturonase-inhibiting protein 2	*P. vulgaris*	Inhibition of fungal endo-polygalacturones	
		*B. napus*	*OsPGIP2*	Polygalacturonase-inhibiting protein 2	*O. sativa*	Inhibition of fungal endo-polygalacturones	[85]
		*B. napus*	*GDSL1*	GDSL lipase	*A. thaliana*	Release of phosphatidic acid from fungal cell membrane and activation of plant defenses	[86]
		*B. napus*	*NPR1*	Non-expressor of pathogenesis-related	*B. napus*	Activation of the SA-mediated plant-defense	[87]
	*Verticillium dahlia*	*B. napus*	*Chit42*	Endochitinase 42	*Trichoderma atroviride*	Fungal cell wall degradation	[61]
	-	*B. juncea*	*ChiC*	Chitinase C	*Streptomyces griseus*	Fungal cell wall degradation	[88]
Oomycetes	*Albugo candida*	*B. napus*	*WRR4*	TIR-NB-LRR protein	*A. thaliana*	Activation of defensive response by JA/ET route	[89]
Nematodes	*Rotylenchulus* sp.	*B. oleracea* var. *capitata*	*Bt*	δ-endotoxin	*Bacillus thuringiensis*	Intestinal toxicity for consumption	[90]
	*Heterodera schachtii*	*B. napus*	*Hs1pro-1*	*Heterodera schachtii* resistance protein	*Beta procumbens*	Activation of a nematode-responsive and feeding site-specific gene expression	[91]
			*cZR3*	CC-NBS-LRR resistance protein		Necrotic hypersensitive response	
Necrotrophic pathogens	-	*B. rapa* ssp. *oleifera*	*entC*	Isochorismate synthase	*E. coli*	SA biosynthesis	[92]

**Table 2:** Transgenic *Brassica* crops against insect and mite pests.

Pest		*Brassica* Species	Gene			Mechanism	Reference
Group	Species		Name	Protein	Origin		
Insects-Hemiptera	*Lipaphis erysimi*	*B. juncea*	*WGA*	Wheat germ agglutinin	Wheat	Not indicated	[103]
		*B. juncea*	*ASAL*	Leaf agglutinin	*Allium sativum*	Blockage of the insect gut epithelial membrane	[104]
		*B. juncea*	*ACA*	Agglutinin	*Allium cepa*	Blockage of the insect gut epithelial membrane	[105]
		*B. juncea*	*Ebf*	(E)-β-farnesene	*Myzus arvensis*	Volatile sesquiterpene compound acting as the main component of aphid alarm pheromones	[106]
		*B. juncea*	*LL*	Lentil lectin	Lentil	Blockage of the insect gut epithelial membrane	[107]
			*CPPI*	Chickpea protease inhibitor	Chickpea	Disruption in assimilation of dietary protein	
		*B. juncea*	*RiD*	Defensin	*Rorippa indica*	Inhibition of nutrient uptake	[108]
		*B. juncea*	*HSPRO2*	Nematode resistance protein-like homolog	*R. indica*	Activation of basal plant resistance	[109,110]
Insects-Lepidoptera	*Helicoverpa armigera*	*B. napus*	*cry1Ac*	Cry1Ac protein	*Bacillus thuringiensis*	Lysis of the gut epithelial cells	[111]
		*B. juncea*	*cry1Ac*	Cry1Ac protein	*B. thuringiensis*	Lysis of the gut epithelial cells	[112]
	*Helicoverpa zea*	*B. napus*	*cry1Ac*	Cry1Ac protein	*B. thuringiensis*	Lysis of the gut epithelial cells	[113]
	*Pieris rapae*	*B. rapa* ssp. *pekinensis*	*cry1C*	Cry1C protein	*B. thuringiensis*	Lysis of the gut epithelial cells	[114]
		*B. rapa* subsp. *pekinensis*	*CpTI*	Cowpea trypsin inhibitor	*Vigna unguiculata*	Inhibition of insect digestive activity	[115]
		*B. napus*	*cry1Ac*	Cry1Ac protein	*B. thuringiensis*	Lysis of the gut epithelial cells	[116]
		*B. oleracea* var. *capitata*	*cry1Ac1*	Cry1Ac1 protein	*B. thuringiensis*	Lysis of the gut epithelial cells	[117]
		*B. oleracea* var. *capitata*	*cry1Ia8*	Cry1Ia8 protein	*B. thuringiensis*	Lysis of the gut epithelial cells	[118]
	*Plutella xylostella*	*B. oleracea* var. *capitata*	*cry1Ab3*	Cry1Ab3 protein	*B. thuringiensis*	Lysis of the gut epithelial cells	[119]
		*B. rapa* ssp. *pekinensis*	*cry1Ab* and *cry1Ac*	Cry1Ab and Cry1Ac proteins	*B. thuringiensis*	Lysis of the gut epithelial cells	[120]
		*Brassica oleracea* var.*italica*	*cry1Ab*	Cry1Ab protein	*B. thuringiensis*	Lysis of the gut epithelial cells	[121]
		*B. rapa* ssp. *pekinensis*	*cry1C*	Cry1C protein	*B. thuringiensis*	Lysis of the gut epithelial cells	[114]
		*B. oleracea* var. *botrytis*	*cryIA(b)*	CryIA(b) protein	*B. thuringiensis*	Lysis of the gut epithelial cells	[122]
		*B. napus*	*cry1Ac*	Cry1Ac protein	*B. thuringiensis*	Lysis of the gut epithelial cells	[123]
		*B. oleracea* var.*italica*	*cryIA(b)*	CryIA(b) protein	*B. thuringiensis*	Lysis of the gut epithelial cells	[124]
		*B. oleracea* var. *acephala*	*cry1Ac* and *cry1C*	Cry1Ac and Cry1C proteins	*B. thuringiensis*	Lysis of the gut epithelial cells	[125]
		*B. oleracea* var. *capitata*	*cry1B* and *cry1Ab*	Cry1B and Cry1Ab proteins	*B. thuringiensis*	Lysis of the gut epithelial cells	[126]
		*B. napus*	*Chi*	Chitinase	*Manduca sexta*	Cuticle degradation	[127]
			*BmkIT*	Insect-specific neurotoxin	*Buthus martensii*	Neurotoxin of contractive paralysis type	
		*B. juncea*	*cry1Ac*	Cry1Ac protein	*B. thuringiensis*	Lysis of the gut epithelial cells	[128]
		*B. juncea*	*cry1Ac* and *cry1C*	Cry1Ac and Cry1C proteins	*B. thuringiensis*	Lysis of the gut epithelial cells	[129]
		*B. napus*	*cry1Ac*	Cry1Ac protein	*B. thuringiensis*	Lysis of the gut epithelial cells	[130]
		*B. oleracea* var. *capitata*	*cry1Ab*	Cry1Ab protein	*B. thuringiensis*	Lysis of the gut epithelial cells	[131]
		*B. juncea*	*cry1C*	Cry1C protein	*B. thuringiensis*	Lysis of the gut epithelial cells	[132]
		*B. napus*	*cry1Ac*	Cry1Ac protein	*B. thuringiensis*	Lysis of the gut epithelial cells	[133]
		*B. napus*	*PjChi-1*	Chitinase 1	*Paecilomyces javanicus*	Cuticle degradation	[76]
			-	Sporamin	Sweet potato	Proteases inhibition	
		*B. oleracea* var. *capitata*	*cry1Ba3*	Cry1Ba3 protein	*B. thuringiensis*	Lysis of the gut epithelial cells	[134]
		*B. juncea*	*cry1Ac*	Cry1Ac protein	*B. thuringiensis*	Lysis of the gut epithelial cells	[135]
		*B. rapa* subsp. *pekinensis*	-	Sporamin	Sweet potato	Disruption in assimilation of dietary protein	[136]
		*B. oleracea* var. *capitata*	*cry1Ia8* and *cry1Ba3*	Cry1Ia8 and Cry1Ba3 proteins	*B. thuringiensis*	Lysis of the gut epithelial cells	[137]
		*B. napus*	cry1C*	Cry1C* protein	*B. thuringiensis*	Lysis of the gut epithelial cells	[138]
		*B. oleracea* var. *capitata*	*cry1Ac1*	Cry1Ac1 protein	*B. thuringiensis*	Lysis of the gut epithelial cells	[117]
		*B. napus*	*cry1Ac*	Cry1Ac protein	*B. thuringiensis*	Lysis of the gut epithelial cells	[139]
		*B. oleracea* var. *capitata*	*cry1Ia8*	Cry1Ia8 protein	*B. thuringiensis*	Lysis of the gut epithelial cells	[118]
		*B. oleracea* var. *capitata*	*cry1Ia8*	Cry1Ia8 protein	*B. thuringiensis*	Lysis of the gut epithelial cells	[140]
		*Brassica oleracea* var.*italica*	*cryIAa*	CryIAa protein	*B. thuringiensis*	Lysis of the gut epithelial cells	[141]
	*Trichoplusia ni*	*B. rapa* ssp. *pekinensis*	*cry1C*	Cry1C protein	*B. thuringiensis*	Lysis of the gut epithelial cells	[114]
		*B. rapa*	*cry1Ac*	Cry1Ac protein	*B. thuringiensis*	Lysis of the gut epithelial cells	[142]
	Not indicated	*B. napus*	*cry1Ac*	Cry1Ac protein	*B. thuringiensis*	Lysis of the gut epithelial cells	[143]
		*B. napus*	*cry1Ac*	Cry1Ac protein	*B. thuringiensis*	Lysis of the gut epithelial cells	[144]
		*B. oleracea* var.*italica*	*cry1Aa*	Cry1Aa protein	*B. thuringiensis*	Lysis of the gut epithelial cells	[145]
Arachnids-Mites	*Tetranychus urticae*	*B. rapa* subsp. *pekinensis*	*COPB2*	Coatomer protein complex subunit 2	*T. urticae*	RNA silencing mechanism	[102]
